# Differential Expression of RBM5, EGFR and KRAS mRNA and protein in non-small cell lung cancer tissues

**DOI:** 10.1186/1756-9966-31-36

**Published:** 2012-04-26

**Authors:** Hong Liang, Jie Zhang, Chen Shao, Lijing Zhao, Wei Xu, Leslie C Sutherland, Ke Wang

**Affiliations:** 1 Department of Respiratory Medicine, Second Affiliated Hospital of Jilin University, Changchun, Jilin, 130041, China; 2 Department of pathophysiology, Norman Bethune College of Medicine of Jilin University, Changchun, Jilin, 130021, China; 3 Department of Respiratory Medicine, Changchun General Hospital, Changchun, Jilin, China; 4 Research Program, Northeast Cancer Centre, Health Sciences North/Horizon Santé-Nord, Sudbury, Ontario, P3E 5J1, Canada

**Keywords:** NSCLC, RBM5, EGFR, KRAS, carcinogenesis

## Abstract

**Background:**

RNA binding motif 5 (RBM5) is a tumor suppressor gene that modulates apoptosis through the regulation of alternative splicing of apoptosis-related genes. This study aimed to detect RBM5 expression in non-small cell lung cancer (NSCLC) and to associate RBM5 expression with clinicopathological data from NSCLC patients and EGFR and KRAS expression to better understand the potential role of RBM5 in NSCLC.

**Method:**

Semi-quantitative reverse transcription-polymerase chain reaction (RT-PCR) and Western blotting were performed to detect expression of mRNA and protein, respectively, of RBM5, EGFR and KRAS in 120 paired non-tumor and tumor samples of NSCLC.

**Results:**

The data showed that expression of RBM5 mRNA and protein was significantly reduced in NSCLC compared to normal tissues, whereas expression of both EGFR and KRAS genes was increased in NSCLC compared to normal tissues. Furthermore, the reduced RBM5 protein expression correlated with smoking status, tumor stage and lymph node metastasis of NSCLC, while overexpression of EGFR and KRAS proteins correlated with tumor stage and lymph node metastasis of NSCLC. Overexpression of KRAS protein was more frequent in smokers with NSCLC. In addition, expression of RBM5 mRNA and protein was negatively correlated with expression of EGFR and KRAS mRNA and protein in NSCLC tissues.

**Conclusion:**

This study suggests further evaluation of RBM5 expression is warranted for use of RBM5 as a biomarker for NSCLC patients.

## Introduction

Lung cancer is a significant worldwide health problem, accounting for more than 1.5 million new cases and 1.3 million cancer-related deaths annually [1,2]. The 5-year survival rate of lung cancer still remains at 13 to 15 % for the past 3 decades, despite recent advances in lung cancer early diagnosis, surgical techniques, and the development of novel chemotherapeutic agents [3]. The single most important risk factor for lung cancer is tobacco smoke, responsible for 85 % of lung cancer incidence. However, lung cancer incidence in developed countries, like several European countries and the USA, was noticeably reduced since 2000, mostly due to tobacco cessation campaigning, whereas the incidence rate in Asian countries, including China and Japan was still shown to be increased [4]. Histologically, lung cancer can be divided into small cell lung cancer and non-small cell lung cancer (NSCLC), which have totally different etiology and treatment options. NSCLC mainly includes squamous cell carcinoma, adenocarcinoma, and large cell carcinoma [5]. Molecularly, NSCLC development is believed to be initiated by the activation of oncogenes or inactivation of tumor suppressor genes [6]. Previous studies demonstrated that mutations in the KRAS proto-oncogene are responsible for 10–30 % of lung adenocarcinomas, while mutations and amplification of EGFR are common in NSCLC and provide the basis for treatment with EGFR-inhibitors [7]. Nevertheless, it remains to be defined how tobacco smoke and other risk factors cause the development of NSCLC, thus, further study of the underlying mechanisms responsible for NSCLC development and progression is truly needed to provide novel strategies in early detection and effective control of this deadly disease.

EGFR, also called HER-1/ErbB1, is a receptor tyrosine kinase (TK) of the ErbB gene family, which contains four closely related proteins, i.e., HER-1/ErbB1, HER-2/neu/ErbB2, HER-3/ErbB3, and HER-4/ErbB4. The EGFR gene is located at chromosome 7p12 and encodes a 170 kDa membrane glycoprotein. Upon binding of specific ligands, such as epidermal growth factor and transforming growth factor-α, the receptor forms homodimers, leading to receptor autophosphorylation and activation of the signal cascade. This results in changes in expression of different genes that are crucial to tumor progression, including tumor growth, resistance to apoptosis, invasion, and angiogenesis [8]. TK activity of EGFR is frequently observed in NSCLC, which maybe dysregulated by several oncogenic mechanisms, including EGFR gene mutation, increased gene copy number, and EGFR protein overexpression [9], as in HER-2, although to a significantly lesser extent [10]. Therefore, targeting of EGFR has achieved significant effects in the clinic; however, elevated EGFR activity is more frequent in never-smokers than smokers, so is less effective in smoking-related lung cancers [11]. In addition, the side effects associated with EGFR targeting necessitate continued research for more specific molecular targets.

KRAS, also known as GTPase KRAS, belongs to the RAS gene family which encodes for a small protein with a molecular weight of 21 kDa with guanosine triphosphatase (GTPase) activity. KRAS acts as a molecular on/off switch. Once it is turned on it recruits and activates proteins necessary for the propagation of growth factors and other receptors' signals, such as c-Raf and PI 3-kinase, involved in many signal transduction pathways [12,13]. The protein product of the normal KRAS gene performs an essential function in normal tissue signaling, and the mutation of a KRAS gene is an essential step in the development of many cancers. Other members of the RAS family include HRAS and NRAS. These proteins all are regulated in the same manner and appear to differ largely by their sites of action within the cell. Previous studies have demonstrated that expression of KRAS was increased in NSCLC, mutations of which were tobacco smoke-related [14]. Although some studies showed that KRAS and EGFR mutations are mutually exclusive and exhibit contrasting characteristics such as clinical background, pathological features of patients, etc., the actual correlation between these two genes and the effective therapeutics for KRAS mutation in NSCLC are still unclear.

RBM5 is one of the approximately 35 genes located in the 370-kilobase tumor suppressor locus on chromosome 3p21.3, loss of which is the most frequent and earliest event in NSCLC [15]. RBM5 plays an important role in the induction of cell cycle arrest and apoptosis through pre-mRNA splicing of multiple target genes, and inhibits tumor transformation and the progression of several malignancies, including NSCLC [16-18]. However, there are only a few studies to date on RBM5 expression in NSCLC.

Our previous study showed that HER2 overexpression was able to downregulate expression of the RBM5 splices variant RBM5 + 5 + 6 in breast cancer cells [19], moreover, RBM5 is downregulated by the constitutively activated RAS mutant protein, RAS(G12V), in rat embryonic fibroblast cells [20], which indicates a correlation between the EGFR and RAS pathways and RBM5 expression. In light of these findings, in this study we set out to examine the expression of RBM5 in NSCLC tissue specimens and the association of RBM5 expression with clinicopathological data and the expression of KRAS and EGFR. This study aims to explore the potential utility of RBM5 as a tumor diagnosis marker in NSCLC.

## Materials and Methods

### Study population

In this study, we collected 120 cases of surgically resected NSCLC and adjacent normal tissues from the Jilin University Affiliated Hospitals between 2008 and 2010. After surgical removal, all of the samples were immediately snap-frozen in liquid nitrogen and stored at −80°C until total RNA was extracted by guanidinium/cesium chloride ultracentrifugation. Patients’ data, including sex, age at diagnosis, tumor histology, clinical stage, and smoking history, were also collected from their medical records. Clinical staging of lung cancers was performed using the revised International System for Staging Lung Cancer [21]. All samples were procured with informed consent after each patient signed the consent form. This study was approved by the Medical Ethics Committee of the First and Second Affiliated Hospital of Jilin University, Changchun, Jilin, China. The detailed outline of the characteristics of our patient cohort is shown in Table 1.

**Table 1:** Association of RBM5, EGFR, and KRAS proteins with clinicopathological characteristics in 120 pair NSCLC specimens

	Total no. of Patients (%)	RBM5			EGFR			KRAS		
		Low(N)	%	P	High(N)	%	P	High(N)	%	P
Characteristic										
**Gender n**										
Male	73(61)	56	76.7	0.46	23	31.5	0.597	34	46.6	0.666
Female	47(39)	28	66.7		18	38.3		20	42.6	
**Age (years)**										
Less than 60	37(31)	26	70.3	0.996	12	32.4	0.586	16	43.2	0.796
Greaterthanorequalto60	83(69)	58	69.7		29	34.9		38	45.8	
**Smoking status**										
Former or Current	84(70)	66	78.6	0.001**	14	38.9	0.475	45	53.6	0.002**
Never	36(30)	18	50		27	32.1		8	22.2	
**Histology, n**										
Adenocarcinoma	47(39)	36	76.6	0.206	19	40.4	0.246	17	36.2	0.119
Squamous cell	73(61)	48	65.8		22	30.1		37	50.7	
**Lymph node Metastasis**										
Positive	60(50 %)	50	83	0.008**	27	45	0.009**	34	56.7	0.01*
Negative	60(50 %)	34	56.7		14	23.3		20	33.3	
**Tumor TNM stage**										
IA	16(13 %)	9	56	0.029**	3	18.7	0.031	2	12.5	0.022*
IB	18(15 %)	11	61		5	27.7		5	27.8	
IIA	28(23 %)	17	60.7		6	35.2		7	25	
IIB	23(19 %)	17	73.9		10	43.5		10	43.5	
IIIA	20(17 %)	17	85		9	45		11	55	
IIIB	15(13 %)	13	86.6		8	53.3		9	60	

(Low) reduced expression patients.(High) increased expression patients. Values represent asymptotic two-tailed significance with asterisks denoting *P < 0.05 and **P < 0.01, from the Pearson’s Chi-squared test.

### Reverse transcription-polymerase chain reaction (RT-PCR)

The expression levels of RBM5, KRAS and EGFR mRNA were determined using a semi-quantitative RT-PCR technique. Briefly, total RNA was isolated from lung tissues using the TRIzol reagent (Invitrogen, Carlsbad, CA, USA) according to the manufacturer's instructions. Reverse transcription was performed with 3 μg of total RNA in a final volume of 10 μl, containing 10 mM dNTP, 0.5 μg oligo dT, 20 U RNasin and 200 U M-MLV reverse transcriptase (Promega Corp., Madison, WI, USA). PCR was performed in a final volume of 25 μl, containing 25 mM MgCl_2_, 2.5 mM dNTP, and 0.5 U Taq DNA polymerase (Invitrogen). PCR amplification was set at an initial 95°C for 5 min and then 28 (GAPDH), 30 (EGFR and KRAS) and 35 (RBM5) cycles of 95°C for 30 s, 55°C for 30s, 72°C for 45 s, and a final extension at 72°C for 10 min. After that, the PCR products were separated by 1 % agarose gel electrophoresis and visualized under UV light after 0.5 % ethidium bromide staining. Gene primers were designed using Primer 5 software (Premier Biosoft International, Palo Alto, CA, USA) and synthesized by Sangong Co. Ltd. (Shanghai, China). The primer sequences were: GAPDH, 5'-GGGTGATGCTGGTGCTGAGTATGT-3' and 5'-AAGAATGGGAGTTGCTGTTGAAGTC-3'; RBM5, 5'-ACACGATG GATGGAAGCCA-3' and 5'-TCTGCTCTGCCTCTGACTT-3'; KRAS, 5'-TCTTGCCTCCCTACCTTCCACAT-3' and 5'-CTGTCAGATTCTCTTGAGCCCTG-3'; EGFR, 5'-TGATAGACGCAGATAGTCGCC-3' and 5'-TCAGGGCACGGTAGAAGTTG-3'.

### Protein extraction and Western blotting

Total cellular protein from lung tissue specimens was extracted according to a previous study [19]. Protein samples (50 μg) were then separated by SDS-PAGE and transferred onto a PVDF membrane (Millipore, Bedford, MA). The primary antibodies were rabbit anti-human RBM5, EGFR and KRAS antibodies from Abcam (MA, USA) and an anti-β-actin antibody from Santa Cruz Biotech, Inc. (Santa Cruz, CA, USA). The secondary antibody was a goat anti-rabbit IgG-HRP from Abcam. Western blotting was carried out as previously described [22], and the protein bands were visualized by SuperSignal West Pico Chemiluminescent Substrate (Pierce, Rockford, IL, USA), and the membranes were subjected to X-ray autoradiography. Band intensities were determined with Quantity One software (Bio-Rad, Hercules, CA, USA). Furthermore, we confirmed the reproducibility of the experiments at least three times. The results were expressed as mean ± S.E.

### Statistical analysis

Pearson’s Chi-squared test was performed to determine the association of clinicopathological data with the expression of RBM5, EGFR, and KRAS mRNA and proteins in NSCLC tissues, and the paired-samples Wilcoxon signed rank test was used to compare the expression of RBM5, EGFR, KRAS mRNA and proteins between NSCLC and adjacent normal tissues. Associations between RBM5, EGFR, and KRAS were tested by using the spearman’s rho test. All statistical analyses were performed using SPSS software, version 17.0. (SPSS, Chicago, IL, USA). A p value equal or less than 0.05 was considered statistically significant. A 2-fold difference between control and test was considered the cut-off point to define over- or under-expression.

## Results

### Differential expression of RBM5 mRNA and protein in NSCLC

**Figure 1:**
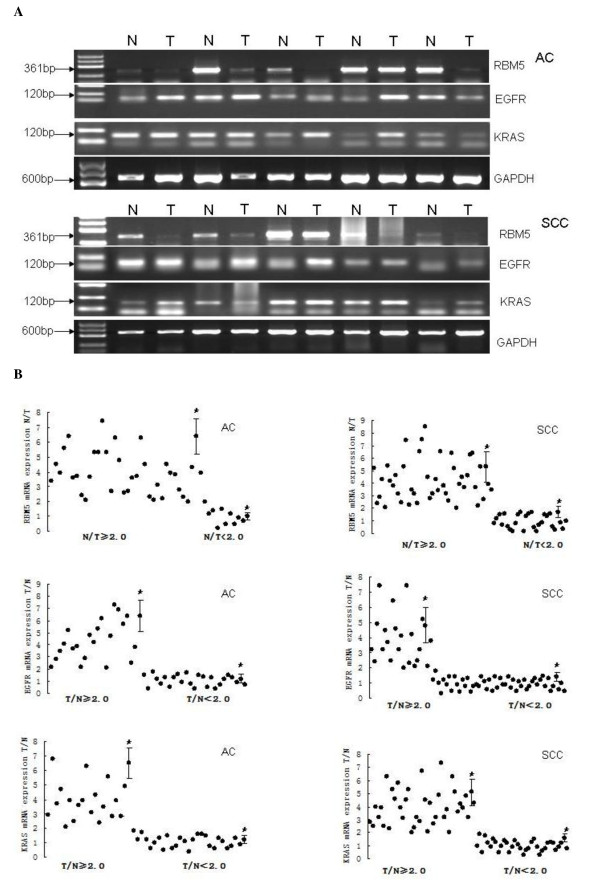
**Expression of RBM5, EGFR and KRAS mRNA in NSCLC**. **A,** Agarose gel of semi-quantitative RT-PCR data of RBM5, EGFR, and KRAS mRNA expression in representative NSCLC and non-tumor specimens. Total RNA was isolated and subjected to semi-quantitative RT-PCR and quantified using Quantity One software. **B**, Quantitative data from A. *p < 0.05 compared to the normal tissues using Wilcoxon signed rank test.

**Figure 2:**
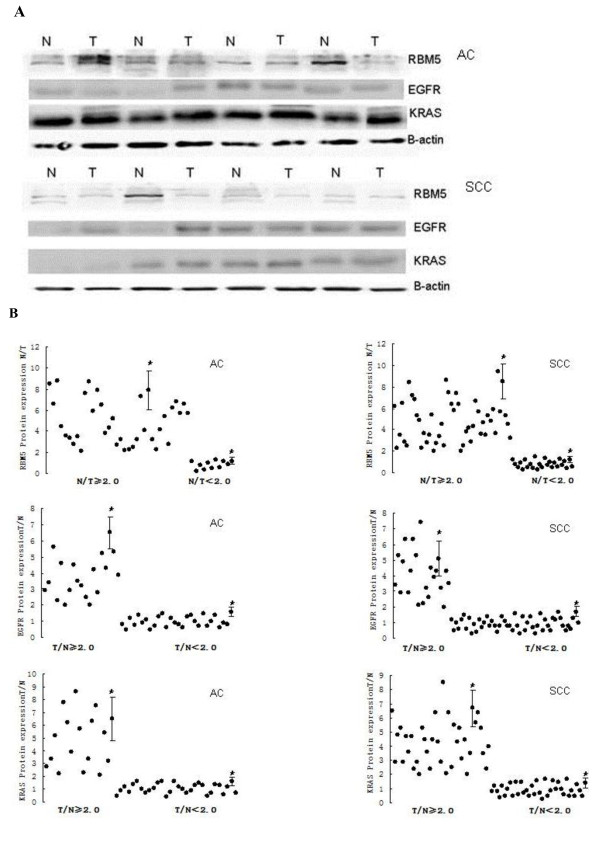
**Expression of RBM5, EGFR and KRAS protein in NSCLC**. **A,** Western blot of RBM5, EGFR and KRAS protein expression in representative tissue samples from NSCLC and non-tumor specimens. Total cellular protein was extracted, subjected to Western blot analysis and quantified using Quantity One software. **B,** Quantitative data from A. *p < 0.05 compared to the normal tissues using Wilcoxon signed rank test.

In this study, we first detected the expression of RBM5 mRNA and protein in 120 paired NSCLC and adjacent normal tissue specimens. Representative data are shown in Figure 1A and Figure 2A. By comparison of normal and tumor expression of RBM5 mRNA and protein at a ratio of 2.0 as a cutoff point we found that expression of RBM5 mRNA and protein was significantly reduced in NSCLC vs. the non-tumor tissues (P = 0.037 and P = 0.03, respectively). Specifically, 78 (65 %) had decreased expression of RBM5 mRNA and 84 (70 %) NSCLC tissues had decreased expression of RBM5 protein. We next examined the association of RBM5 protein expression with the clinicopathological data for the NSCLC patients and found that the decreased expression of RBM5 protein was significantly more frequent in smokers than in non-smokers (66 vs. 18 cases or 78.6 % vs. 50 %; P = 0.001). Reduced RBM5 protein expression in the NSCLC tissues was also significantly positively correlated with lymph node metastasis of NSCLC patients (50 vs. 34 or 83 % vs. 56.7 %; P = 0.008). RBM5 protein expression also associated with tumor stages. Decreased RBM5 protein expression was more frequently observed in NSCLC patients with IIIA and III B stages compared to those with I and IIA stages (Table 1).

### Differential expression of EGFR mRNA and protein in NSCLC

Next, we analyzed the expression of EGFR mRNA and protein in 120 cases of NSCLC and adjacent normal tissue specimens. The data are summarized in Figure 1A and Figure 2A. By comparison of normal and tumor expression of EGFR mRNA and protein at a ratio of 2.0 as a cutoff point, we found that expression of EGFR mRNA and protein was significantly increased in NSCLC tissues compared the non-tumor tissues (P = 0.024 and P = 0.008, respectively). Specifically, increased expression of EGFR mRNA was found in 45 (37.5 %) tumor tissues, while the increased expression of EGFR protein was found in 41 (34.2 %) tumor tissues. In lung adenocarcinoma, the increased expression of EGFR protein was found in 19 (40.4 %) tumor cases and, in squamous cell carcinoma, 22 (30.1 %) cases had overexpressed EGFR protein (P = 0.246). Furthermore, we found that the increased expression of EGFR protein was more frequent in lymph node metastasis of NSCLC compared to non-metastatic NSCLCs (27 vs. 14 or 45 % vs. 23.3 %; P = 0.009). Expression of EGFR protein also associated with tumor stages. Increase EGFR protein expression was more frequently observed in patients with IIIA and IIIB compared to those in I and IIA. But there was no association of EGFR expression with other clinicopathological data from NSCLC patients (Table 1).

### Differential expression of KRAS mRNA and protein in NSCLC

Expression of KRAS mRNA and protein in 120 cases of NSCLC and adjacent normal tissue specimens is summarized in Figure 1A and Figure 2A. By comparison of normal and tumor expression of KRAS mRNA and protein at a ratio of 2.0 as a cutoff point, we found that expression of KRAS mRNA and protein was significantly increased in NSCLC compared the non-tumor tissues (P = 0.03 and P = 0.018, respectively). Specifically, increased expression of KRAS mRNA was found in 52 (43 %) tumor tissues, while the increased expression of KRAS protein was found in 54 (45 %) tumor tissues. Moreover, the increased expression of KRAS protein was found in 17 (36.2 %) adenocarcinoma samples and in 37 (50.7 %) squamous cell carcinoma samples. Increased expression of KRAS protein was more frequent in squamous cell carcinomas and in lymph node metastasis compared to non-metastatic tumors (34 vs. 20 or 56.7 % vs. 33.3 %; P = 0.01). Expression of KRAS protein was associated with tumor stages and also occurred more frequently in ever-smokers (P = 0.002; Table 1).

### RBM5, EGFR and KRAS expression correlations in NSCLC

We examined the relationship between expression of RBM5, EGFR, and KRAS in NSCLC and found that expression of RBM5 mRNA and protein was significantly negatively correlated with expression of EGFR and KRAS mRNA and protein in NSCLC tissues (p < 0.01; Tables 2 and 3).

**Table 2:** Association of RBM5 with EGFR and KRAS mRNA expression

	EGFR-T	KRAS-T
RBM5-T		
Correlation coefficient	−0.961	−0.809
Sig.(2-tailed)A	0.000**	0.000**
N	120	120

^a^P-values represent asymptotic two-tailed significance with asterisks denoting **P < 0.01, from the Spearman`s rho test.

**Table 3:** Association of RBM5, EGFR, and KRAS proteins expression

	EGFR-T	KRAS-T
RBM5-T		
Correlation coefficient	−0.943	−0.842
Sig. (2-tailed)A	0.000**	0.000**
N	120	120

^a^P-values represent asymptotic two-tailed significance with asterisks denoting **P < 0.01, from the Spearman`s rho test.

## Discussion

In this study, we analyzed the expression of RBM5, EGFR, and KRAS genes in NSCLC and adjacent normal tissue specimens and found that RBM5 expression was reduced in NSCLC compared to the normal tissues, whereas expression of both EGFR and KRAS genes was increased in NSCLC compared to the normal tissues. The reduced expression of RBM5 protein was associated with tobacco smoke, tumor stages, and lymph node metastasis of NSCLC, while overexpression of EGFR and KRAS proteins was associated with tumor stages and lymph node metastasis of NSCLC. Overexpression of KRAS protein occurred more frequently in smokers with NSCLC. Moreover, expression of RBM5 mRNA and protein was negatively associated with expression of EGFR and KRAS mRNA and protein in NSCLC tissues. The data from the current study suggest that expression of RBM5 mRNA and protein is worth further evaluation as a biomarker for tumor diagnosis.

Previous studies have shown that RBM5 expression was frequently reduced in different cancers, including breast cancer [20], human schwannoma [23] and 75 % of primary lung cancer specimens [24]. In the present study, expression levels of RBM5 protein were reduced in NSCLC compared with the non-tumor tissues, suggesting that RBM5 could play a role in suppression of NSCLC development or progression. Furthermore, the expression level of RBM5 was shown to be high in the adult thymus and low in the fetal thymus, indicating that RBM5 expression may be developmentally regulated [17]*.* RBM5 protein is a negative regulator of cell proliferation: overexpression of the full length LUCA-15/RBM5 in breast cancer CEM-C7 and NSCLC A549 cells suppressed cell proliferation through induction of apoptosis and arrest of tumor cells at the G1 phase of the cell cycle [16]. These data together suggest that the loss of RBM5 expression in different cancer tissues and cells contributes to tumor growth via regulation of cell proliferation and apoptosis.

Moreover, our current study also showed that expression of RBM5 protein in NSCLC tissues was negatively correlated with tobacco smoke, The data that decreased expression of RBM5 protein was more frequent in smokers than in non-smokers suggest tobacco carcinogens may lead to the loss of RBM5 expression in NSCLC, which is in agreement with previous studies that had shown deletions at 3p21.3 were the earliest lesions in lung cancer, and were associated with smoking alone [15].

In addition, tumor metastasis, the major cause of cancer death, is a multistep process that requires interactions between cancer cells, stromal cells, and the extracellular matrix. In this study, we found that reduced expression of RBM5 protein was associated with lymph node metastasis of NSCLC, indicating that RBM5 may play a potential role in the suppression of tumor metastasis. It is further corroborated by other studies, including (1) RBM5 downregulation as a part of a molecular signature of 17 genes for detection of metastasis of multiple solid tumor types [25,26]: solid tumors with these gene signatures had high metastasis rates and poor clinical outcomes, and (2) demonstration that RBM5 may regulate inhibition of metastasis in lung cancer through the upregulation of some metastasis-related genes including Rac1, B-catenin, collagen and laminin [27]. Taken together, in the light of all the observations, we suggest that RBM5 could be a promising candidate towards lung cancer clinical management in terms of the metastatic status. Nevertheless, the detailed molecular mechanism involved in RBM5-mediated metastasis needs to be further investigated.

Our data also showed an inverse correlation between RBM5 expression and EGFR and KRAS expression in NSCLC. Alteration of EGFR expression and gene amplification has been reported as between 7 % and 45 % in lung cancer cases [28-30], which may also be due to variations in techniques, criteria to determine positivity, and inter-observer variability [29,30]. In our study, overexpression of EGFR was found in 33 % of specimens of NSCLC, with a somewhat higher incidence in ACs than in SCCs. Moreover, overexpression of KRAS was found in 45 % of specimens of NSCLC, with a somewhat higher incidence in SCCs than in ACs. Overexpression of EGFR and KRAS proteins was associated with lymph node metastasis and with a more advanced pathologic stage. Our current study for the first time demonstrated a correlation between the expression levels of RBM5, EGFR and KRAS in NSCLC tissues, with the data suggesting that disruption of RBM5 apoptosis-induced activity and tumor suppressor function is consistent with the potent oncogenic activity associated with EGFR and KRAS overexpression. The differential expression of these three genes in NSCLC suggests the presence of a complex regulatory network involving tumor suppression and oncogenic expression.

Details of the inverse relationship between RBM5, EGFR and KRAS are only beginning to be delineated [19,31]. For instance, HER2 overexpression was shown to affect the alternative splicing of RBM5. One cytotoxic isoform, RBM5 + 5 + 6 t, was downregulated in breast cancer cells (both primary tumors and a cell line) that have overexpressed HER2 [19], which suggested that factors in the EGFR pathway may function as upstream modulators of RBM5 function and/or expression. In order to investigate this hypothesis, we downregulated EGFR in NCI-H1975 lung adenocarcinoma cells that have activated EGFR, using small interfering RNA, and analyzed RBM5 expression [CMJ, submitted]. The results of this study demonstrated that downregulation of activated EGFR, in the NCI-H1975 lung cancer cell line, did not, in fact, correlate with upregulation of RBM5, suggesting that RBM5 functions upstream of EGFR. That deletion of the region encompassing the RBM5 gene is one of the earliest lesions associated with smoking does suggest that downregulation of RBM5 is necessary for cancer initiation events. In addition, we recently observed that overexpression of RBM5 induced expression of KRAS in the A549 lung cancer cell line and decreased expression of KRAS in the MCF-7 breast cancer cell line [Wang et al, unpublished data], suggesting that RBM5 functions upstream of KRAS, albeit with different outcomes, depending on tissue of origin.

In conclusion, our study suggests that further study of RBM5, EGFR and KRAS gene function and inter-relationships will provide a better understanding of the role these genes play in NSCLC development and progression.

## Abbreviations

NSCLC = Non-small cell lung cancer; RBM5 = RNA Binding Motif 5; EGFR = Epidermal growth factor receptor; KRAS = GTPase KRAS.

## Misc

Hong Liang and Jie Zhang contributed equally to this work

## Competing interests

The authors declare that they have no competing interests.

## Authors’ contributions

HL performed all the experiments and drafted the manuscript. CS and LZ participated RNA and protein extraction. WX collected and provided the tissues. JZ and KW have contributed the research design, the data collection and interpretation. KW oversaw the design of the study, was involved in the critically revised the manuscript. LCS oversaw the manuscript and gave a thorough revision. All authors have read and approved the final version of the manuscript.
